# Poly(vinyl pyridine) and Its Quaternized Derivatives: Understanding Their Solvation and Solid State Properties

**DOI:** 10.3390/polym14040804

**Published:** 2022-02-19

**Authors:** Katerina Mavronasou, Alexandra Zamboulis, Panagiotis Klonos, Apostolos Kyritsis, Dimitrios N. Bikiaris, Raffaello Papadakis, Ioanna Deligkiozi

**Affiliations:** 1 Creative Nano PC, 4 Leventi Street, Peristeri, 12132 Athens, Greece; k.mavronasou@creativenano.gr; 2 Laboratory of Polymer Chemistry and Technology, Department of Chemistry, Aristotle University of Thessaloniki, 54124 Thessaloniki, Greece; azampouli@chem.auth.gr (A.Z.); pklonos@central.ntua.gr (P.K.); dbic@chem.auth.gr (D.N.B.); 3 Department of Physics, Zografou Campus, National Technical University of Athens, 15780 Athens, Greece; akyrits@central.ntua.gr; 4 TdB Labs AB, Ulls Väg 37, 75651 Uppsala, Sweden; rafpapadakis@gmail.com

**Keywords:** poly(4-vinylpyridine), poly(N-methyl-4-vinylpyridinium iodide), quaternization, solvatochromism, preferential solvation, optical energy gap

## Abstract

A series of N-methyl quaternized derivatives of poly(4-vinylpyridine) (PVP) were synthesized in high yields with different degrees of quaternization, obtained by varying the methyl iodide molar ratio and affording products with unexplored optical and solvation properties. The impact of quaternization on the physicochemical properties of the copolymers, and notably the solvation properties, was further studied. The structure of the synthesized polymers and the quaternization degrees were determined by infrared and nuclear magnetic spectroscopies, while their thermal characteristics were studied by differential scanning calorimetry and their thermal stability and degradation by thermogravimetric analysis (TG-DTA). Attention was given to their optical properties, where UV-Vis and diffuse reflectance spectroscopy (DRS) measurements were carried out. The optical band gap of the polymers was calculated and correlated with the degree of quaternization. The study was further orientated towards the solvation properties of the polymers in binary solvent mixtures that strongly depend on the degree of quaternization, enabling a better understanding of the key polymer (solute)-solvent interactions. The assessment of the underlying solvation phenomena was performed in a system of different ratios of DMSO/H_2_O and the solvatochromic indicator used was Reichardt’s dye. Solvent polarity parameters have a significant effect on the visible spectra of the nitrogen quaternization of PVP studied in this work and a detailed path towards this assessment is presented.

## 1. Introduction

Quaternized nitrogen involving polymers are a class of highly functional macromolecules that exhibit the properties of conventional polymers and an enhanced sensitivity towards the external environment due to the existing charges, e.g., solvent polarity, pH [1,2]. They can be classified in the broader class of polyelectrolytes (PEL) [3], displaying dual behavior and combining the properties of both polymers and electrolytes [4,5,6]. Unique properties are derived from their distinct nature, such as excellent water solubility, strong binding interactions with oppositely charged surfaces and molecules as well as a notable solvation and swelling behavior. Due to these features, they are widely employed as viscosity and surface modifiers as well as selective adsorbers. Consequently, these materials are useful in various industrial applications, materials and formulations from water treatment [7], membranes [8,9], sensors [10] and cosmetics to biomedicine with specific uses in bacterial and viral infections [11,12,13,14,15,16,17,18,19].

Poly(4-vinylpyridine) (PVP) is a linear polymer with pendant pyridine groups that can be used in a variety of applications, such as surface modification, by immobilizing atoms or particles, electrochemical sensors [20], fabrication of antibacterial surfaces, development of pH sensitive systems, 3D molecular level ordering systems, anti-corrosive coatings and even dye sensitized solar cells (DSSCs) and light-emitting diodes [21,22,23,24,25,26,27,28,29]. Positively charged polypyridines constitute a unique class of compounds in which quaternization enables the introduction of permanent positive charges into the backbone, resulting in electron delocalization phenomena that are attributed to the existence of electron-rich pyridines and electron-poor pyridiniums [30,31,32,33]. As a result, these polymers exhibit improved molecular properties, such as non-linear optical activity and increased conductivity [5,12,18,34,35,36]. Moreover, the counterion, as well as the length of the alkyl chain of the methylating agent, have a significant influence on solubility and conductivity [37,38,39]. Counterion exchange has been studied in order to obtain specific properties. It should be noted that Reillex ion exchange resins are commercial resins based on crosslinked and partially quaternized poly(4-vinylpyridine). Finally, amphiphilic copolymers containing a quaternized PVP block have also been reported [40,41,42].

Although it has been observed that 4-vinylpyridine polymerizes spontaneously upon protonation/quaternization, affording quaternized PVPs [43,44]; in the present study, quaternization was performed by a post-polymerization step, a strategy that yields well-defined polymers more readily [45,46]. Quaternized PVPs with a variety of alkyl halides have been reported, with various alkyl lengths and counterions. Most quaternization reactions afford quaternization degrees around 70%, while quantitative quaternizations have also been reported, usually achieved owing to a large excess of alkyl halide [14,15,17,47,48]. Although the kinetics of quaternization reactions and the retardation effect observed have been studied extensively ([49,50,51] and references therein), to the best of our knowledge, a systematic investigation on structure/properties relationship, especially focused on solvation and optical properties, regarding partially quaternized PVP derivatives is lacking. Indeed, most previous works focus on the use of a highly quaternized PVP derivative for specific applications, while the more fundamental study of the impact of quaternization on the molecular structure and resulting behavior of quaternized PVPs has not been reported yet.

**Scheme 1:**
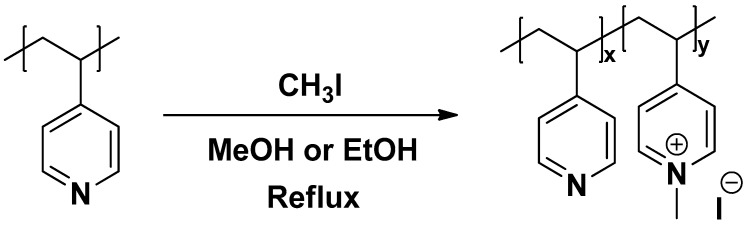
Illustration of the synthetic route for the fully quaternized (x = 0) and the partially quaternized derivatives.

In the present study, we report the targeted synthesis and characterization of fully (PVPQ) as well as three partially quaternized derivatives, produced by N-alkylation of the pyridine ring with methyl iodide. The aim is to cover a full range of quaternization degrees and perform a comparative study of the impact of quaternization on the physicochemical and optical properties as well as the solvation behavior of the resulting derivatives. Poly(4-vinylpyridine) (PVP) was quaternized in the presence of methyl iodide according to Scheme 1. The quaternization was quantified by ^1^H NMR spectroscopy. The response of the polymers to temperature was assessed via TGA and DSC analysis. Emphasis was given on their behavior when it comes to light absorbance, reflectance and solvent interactions. Significant differences were observed between neutral PVP and the fully quaternized derivative (PVPQ), while the partially quaternized polymers displayed an intermediate behavior. The optical band gap difference and the solvation effect in binary solvent mixtures between those two polymers prove the significance of quaternization.

## 2. Materials and Methods

### 2.1. Materials

Poly(4-vinylpyridine) (Mw = 60 kDa) (PVP) and methyl iodide (CH_3_I, 99.5%) were used as received from Sigma-Aldrich (St. Louis, MO, USA). Methanol (MeOH, Read, Ph.Eur) and ethanol (EtOH, 99.8%) were supplied by AppliChem GmbH (Darmstadt, Germany) and dimethyl sulfoxide (DMSO) A.G by Penta (Prague, Czech Republic). Reichardt′s dye (dye content 90%) (RB) from Sigma-Aldrich (St. Louis, MO, USA) was used as the solvatochromic indicator.

### 2.2. Synthesis of Poly(N-methyl-4-vinyl pyridinium iodide) Derivatives

In this study, a fully quaternized (PVPQ) and three partially quaternized derivatives (PVP_Q1-3) (Table 1), were synthesized. The polyvinyl pyridine/methyl pyridinium copolymers were synthesized by dissolving 1 g (0.0167 mmol) of poly(4-vinylpyridine) (PVP) in 100 mL of ethanol and refluxing the solution in the presence of 0.35 mL (0.0055 mol), 0.47 mL (0.007 mol) and 0.7 mL (0.011 mol) of methyl iodide for 4 h so that, PVP_Q3, PVP_Q2, and PVP_Q1 were synthesized, respectively [17]. For the PVPQ derivative, 1 g of poly(4-vinylpyridine) (0.0167 mmol) was added to 50 mL of methanol and the solution was refluxed in the presence of 0.9 mL (0.014 mol) of iodomethane for 4 h. All the synthesized polymers were precipitated from the respective reaction mixtures using diethyl ether (200 mL approx.), filtered, and washed with ethanol and diethyl ether to yield a yellowish/greenish polymer product. All the resulting products were dried and fully characterized without further treatment.

The reaction yields for PVP_Q3, PVP_Q2, PVP_Q1 and PVPQ were 63%, 71%, 99.8% and 84%, respectively. The yields were calculated by comparing the initial PVP moles with the moles of the final product after precipitation and drying. The molecular weight of the initial PVP is 60 kDa, i.e., approximately 570 repeating units. The molecular weight of each newly synthesized polymer was calculated based on the degree of quaternization found by ^1^H NMR (see Equation (1)), on the basis of 570 repeating units and a Mw of 247 g/mol per quaternized repeating unit. 

### 2.3. Characterization Methods

The Fourier transform infrared (FTIR) spectra of the synthesized samples were measured on a Brucker Tension 27 FTIR spectrometer (Karlsruhe, Germany), equipped with a diamond ATR accessory at 25 °C and a spectral resolution of 4 cm^−1^ in the 4000–600 cm^−1^ range. IR spectra were analyzed with the Bruker OPUS software (version 5.2).

Nuclear magnetic resonance (NMR) spectra were recorded on an Agilent spectrometer (Agilent AM 600, Agilent Technologies, Santa Clara, CA, USA), operating at a frequency of 600 MHz for protons. Deuterated water, methanol or their mixtures were used depending on the solubility of the polymers. The spectra were internally referenced with tetramethylsilane (TMS) and calibrated using the residual solvent peak. 

X-ray powder diffraction (XRD) was employed to study the structure of the synthesized polymers. The XRD spectra were recorded at room temperature through a MiniFlex II XRD system (Rigaku Co., Tokyo, Japan), with Cu Ka radiation (*λ* = 0.154 nm), over the 2θ range from 5° to 60° with a scanning rate of 1 °/min.

Differential scanning calorimetry (DSC) measurements were carried out in the overall temperature range from –40 to 210 °C in high purity nitrogen (99.9995%) atmosphere, utilizing a TA Q200 series DSC instrument (TA, New Castle, DE, USA), calibrated with indium for temperature and enthalpy and sapphires for heat capacity. The measurements were performed on samples of ~8–9 mg in mass closed in TA aluminum Tzero Hermetic pans. The cooling and heating rates were fixed at 10 °C/min. Overall, four (4) scans were performed in DSC, in order to follow effects on the samples as received, namely, equilibrated at environmental conditions, and upon drying. Details on the thermal profiles are given below, along with the experimental results.

The thermal stability analysis of the polymers was carried out under inert atmosphere (N_2_) and air (N_2_ 80% O_2_ 20%). The measurements under air were performed using a thermogravimetric analyzer PerkinElmer Diamond Thermogravimetric/Differential Thermal Analysis (TG/DTA) (Waltham, MA, USA). The gas flow rate was 80 mL/min. The specific operation steps were as follows: the samples were heated from 25 to 700 °C at a rate of 10 °C/min and were kept at 7000 °C for 5 min. Thermogravimetric analysis under nitrogen was performed with a SETARAM SETSYS TG-DTA 16/18 instrument (Setaram instrumentation, Lyon, France). Samples were heated from ambient temperature to 600 °C in a 50 mL/min flow of N_2_. A nominal heating rate of 20 °C/min was used and continuous records of sample temperature, sample weight, and heat flow were taken. All measurements were performed in triplicate.

UV-Vis spectra in solution and diffuse reflectance spectra (DRS) in the solid-state were measured between 200–800 nm using an Agilent Carry 60 spectrometer. UV-Vis spectra of PVP and its quaternized derivatives were measured in methanol and demonstrated characteristic bands at a concentration of 50 ppm. All measurements were performed at 25 ± 1 °C. The slit width and data interval were set to 1 nm, and the scan speed in all measurements was 960 nm/min. All spectra were normalized to show the same maximum intensity.

The DRS measurements were performed using a DRA Fibre Optic coupler (Harrick Agilent Barrelino). The bandgap was calculated using the Kubelka-Munk (K-M) model by plotting [F(R) × E]^1/2^ vs. E (eV), where F(R) = (1 − R)^2^/2R is the K-M function and R is the reflectance of the materials. 

### 2.4. Solvatochromic Properties Study

The solvatochromic study was carried out using the following protocol: Firstly, 130 and 150 ppm stock solutions of the polymers in water (PVPQ) and ethanol (PVP) were prepared, respectively, and 0.1 mL of each solution was left to evaporate overnight in several vials. A Reichardt′s dye stock solution was also prepared in acetone (150 ppm) and 1 mL of it was also left to evaporate overnight and dried under vacuum the next day. Then, to each polymer-containing vial, 1 mL of each binary solvent mixture ratio was added and after dissolution, the solvents were left to equilibrate with the polymer for 2 h. The binary mixtures of DMSO and water were prepared in multiple ratios in order to cover in detail the variation of the polymers behavior in the presence of slightly different solvent polarities. The same solvent mixture ratio was added to both polymers each time so that a comparative study would be performed. The UV spectra of each polymer-binary mixture was measured and used as a baseline for the measurement of the RB. After transferring the aforementioned quantity to the dye containing vials and dissolving the dye, RB’s λmax was recorded.

## 3. Results

### 3.1. Polymer Synthesis and Structural Characterization

**Figure 1:**
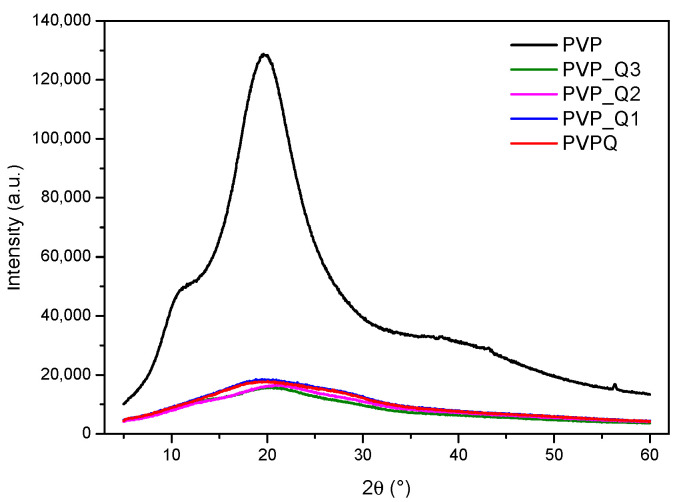
X-ray diffraction patterns of the synthesized polymers.

Poly(4-vinylpyridine) (PVP) was quaternized in the presence of methyl iodide according to Scheme 1. Progressively increasing amounts of methyl iodide afforded increasing quaternization degrees (up to 100%). Methanol was used as a solvent for the synthesis of the fully quaternized polymer (PVPQ), while ethanol was preferred for the partially quaternized derivatives (PVP_Q1-3). The success of these reactions was confirmed by FTIR and NMR spectroscopy, as discussed below. According to XRD measurements the obtained polymers were amorphous, Figure 1.

**Figure 2:**
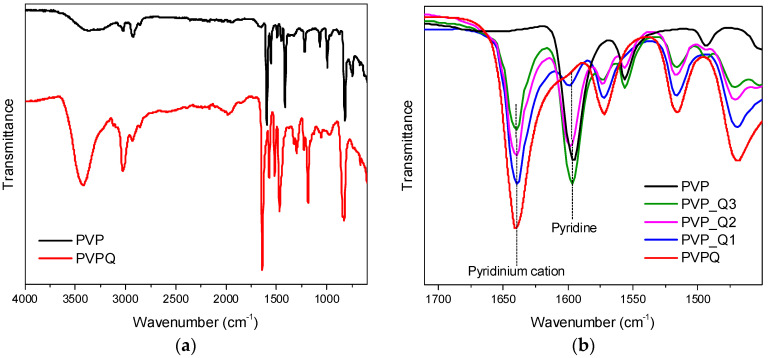
(**a**) Full FTIR spectra of PVP vs. the fully quaternized PVPQ derivative; (**b**) FTIR spectra of the 1400–1700 cm^−1^ region of the quaternized derivatives vs. the non-quaternized PVP.

The FTIR spectrum of PVP in Figure 2a exhibits the characteristic vibrations of the pyridine ring (C=C, C–N) at 1598, 1556, 1496, 1452 and 1415 cm^−1^ [18]. The absorption bands at 1068 and 957 cm^−1^ can be assigned to the in-plane and out-of-plane C–H bending, respectively. The C-H stretching bands of the chain and the pyridine pendants are observed in the 3000 cm^−1^ region. For PVPQ, the appearance of the pyridinium bands at approximately 1640, 1570 and 1516 cm^−1^ confirm the quaternization of PVP [23,43]. It must also be noted that there is no trace of PVP, as evidenced by the absence of a band at 1598 cm^−1^. The bands that appear at 1638, 1570, 1469 and 1300 cm^−1^ correspond to the C-C and C-N stretching vibrations of the pyridinium cation. The other peaks at 1185, 1056 and 971 cm^−1^ correspond to the symmetric bending of CH_3_, the stretching of CH_3_–N, and the out-of-plane stretching of H–C bond, respectively.

The PVP_Q derivatives exhibit both peaks attributed to PVP and PVPQ. More specifically in Figure 2b, the peak at 1598 cm^−1^ corresponding to the non-methylated pyridine weakens when the quaternization ratio increases, while the peak attributed to the quaternized pyridine units at 1640 cm^−1^ increases accordingly. Last but not least, the peak around 3400 cm^−1^ in the full spectra is gaining intensity in the case of PVPQ when compared to PVP, due to the higher hydrophilicity and consequently, larger amount of moisture bound to the polymer [52].

**Figure 3:**
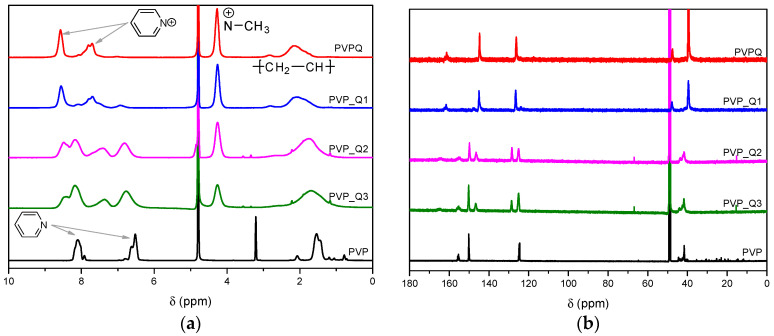
(**a**) ^1^H NMR and (**b**) ^13^C NMR spectra of poly(4-vinylpyridine) (PVP), partially (PVP_Q1–PVP_Q3) and fully (PVPQ) quaternized polymers.

The IR results are corroborated by NMR spectroscopy (Figure 3). In the ^1^H NMR spectra, Figure 3a, the broad resonance signal around 1.5–2 ppm corresponds to the aliphatic protons of the backbone (–CH_2_–CH–). In the quaternized polymers, the methyl group is clearly observed at ca. 4.2 ppm. The intensity of this peak increases with the quaternization degree. In the aromatic region, the protons of the pyridine and pyridinium aromatic rings are observed at 6.6 and 8.2 ppm, and 7.7 and 8.5 ppm, respectively. Similarly to the peak at 4.2 ppm, the resonance signals corresponding to the pyridinium units increase as the quaternization degree increases. Finally, in PVPQ, the peaks corresponding to PVP have completely disappeared confirming the quantitative quaternization of PVP [47,53]. Similar observations are drawn from the ^13^C NMR spectra, Figure 3b: the resonance signals attributed to PVP (155, 150 and 124 ppm) decrease as the quaternization degree increases and are totally missing from PVPQ spectra. Correspondingly, the signals corresponding to the quaternized rings (160, 147 and 128 ppm) progressively increase.

^1^H NMR spectra were used to calculate the quaternization degree. Due to the broadness of the peaks and some overlapping, the ratio of pyridine to pyridinium units could not be calculated by directly comparing the integrations of the corresponding peaks. It was thus calculated indirectly, according to Equation (1). On one hand, it is assumed that the integration of the peaks from 6–8 ppm corresponds to the total amount of units (quaternized and non-quaternized). On the other hand, when the peak corresponding to the methyl group at 4.2 ppm is normalized to three for all derivatives, the amount of quaternized units is equal to the amount of quaternized units in PVPQ, i.e., 4.1. The “excess” of aromatic protons is attributed to non-quaternized pyridine units. The ratio of those two integrations gives the degree of quaternization. The calculated quaternization degrees are reported in Table 1.

(1) $$Quaternization\;degree\;\left(\%\right)=\frac{I{\left(6-8\right)}_{PVPQ}}{I{\left(6-8\right)}_{PVP\_Qx}}\times 100\;$$

where *I(6−8)_PVPQ_* is the integration of the aromatic protons in for PVPQ, i.e., 4.1, and *I(6−8)_PVP_Qx_* is the total integration of the aromatic protons for the partially quaternized derivatives.

### 3.2. Thermal Properties and Stability

The thermal behavior of PVP and all PVPQ derivatives was studied by differential scanning calorimetry (DSC). Four scans were performed by DSC. A first heating scan (scan 1), up to 150 °C, was performed to erase the thermal history in the presence of hydration water, and, subsequently (scan 2), the samples were cooled to a low temperature and heated up to 160 °C, in order to simultaneously follow the effects of structure and hydration on glass transition. Then, for scans 3 and 4, holes were made in the upper side of the hermetic pans to allow water evaporation. The samples were heated up to 160 °C (scan 3, water evaporation) and, subsequently cooled and heated up to 210 °C (scan 4). Thus, during scan 4 the direct effects of structure on glass transition could be assessed.

**Figure 4:**
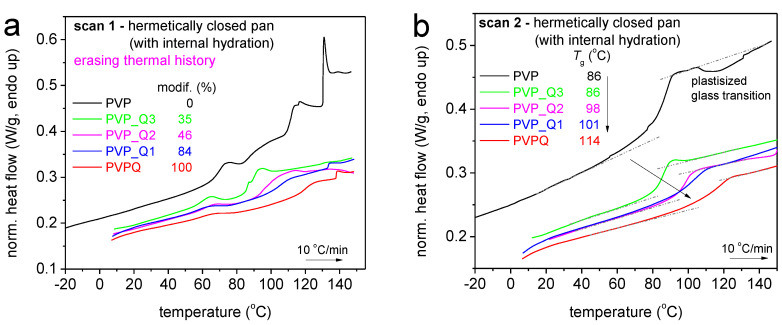
Heat flow curves during (**a**) scan 1 and (**b**) scan 2. The heat flow has been normalized to the sample mass. The glass transition temperatures, Tg, have been added to the plot. The added dash-dotted lines represent the baselines prior and upon glass transition.

**Figure 5:**
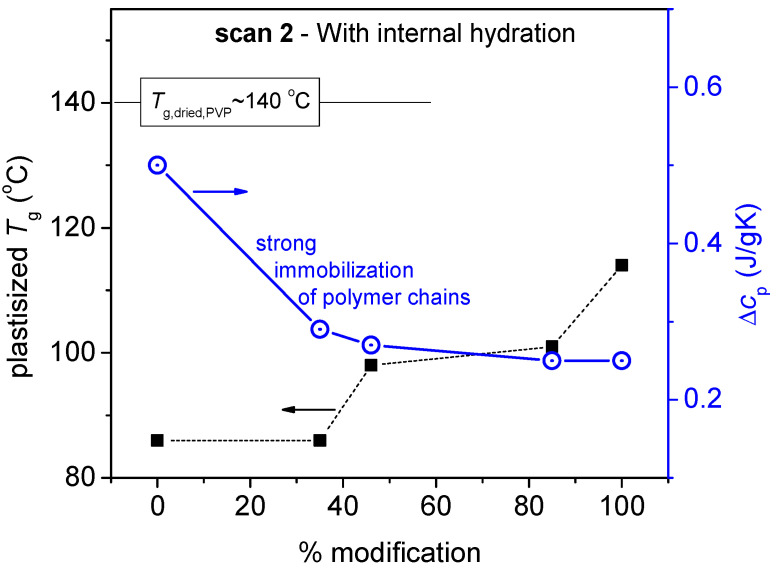
The modification effect on (left axis) Tg and (right axis) glass transition strength, Δc_p_, in the ambient hydrated samples (as received). Included is the Tg for the initial dried PVP.

During scan 1 in DSC, all samples exhibit complex endothermal phenomena at temperatures above the glass transition (Figure 4a). Upon the erasing of thermal history and fixing of the polymer-pan thermal contact by the first heating, we may observe in Figure 4b that all samples demonstrate single glass transition steps. From a glance, it seems that the quaternization leads to elevation of the characteristic glass transition temperature, Tg (Table 1), and suppression of the glass transition strength (or else change in the heat capacity), Δc_p_. These values were estimated and are shown in Figure 5 as a function of quaternization/modification. Beginning with PVP, which exhibits a Tg of 86 °C and Δc_p_ of 0.50 (±0.01) J/gK, the modification results in a sharp increase in Tg and decrease in Δc_p_ from the lower modification level. The changes are monotonic and suggest that the quaternization severely affects the mobility of polymer chains and particularly hinders the polymer chain diffusion (increase in Tg) and suppresses the mobile amorphous chains fraction (decrease of Δc_p_) [54,55,56,57]. Both results are indicative of the transformation of the flexible polymer matrix (PVP) to a significantly more rigid one. This can also be observed from a qualitative effect in Figure 4b, namely, the change of heat flow slope (baseline) from large to gradually lower, both prior and upon glass transition [54,55]. 

Besides these recordings, we should keep in mind that the abovementioned values refer to hydrated samples. Based on the dehydration experiments of scan 3 and 4, the Tg of the dried PVP equals ~140 °C (Figure 4). Therefore, it is essential that both the PVP and the quaternized PVPs are plasticized, i.e., exhibit lower Tg via the increased free volume involved due to the presence of water. Then, the elevated Tg in the hydrated PVPQ and PVP_Q1-3 should originate from the synergetic effect of hydration water and quaternization. 

**Figure 6:**
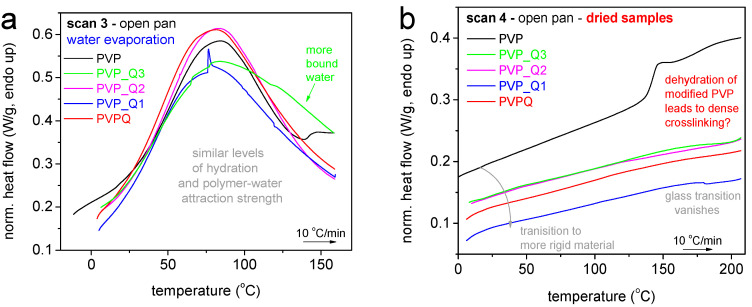
Comparative heat flow curves during heating for (**a**) scan 3 and (**b**) scan 4. The heat flow has been normalized to the sample mass.

In Figure 6a, we present the DSC results for scan 3. The endothermic peak recorded between RT and 150 °C corresponds to the evaporation of ‘free’ and ‘semi-bound’ water [58]. The position of the peak (85–90 °C) barely changes between the different samples, whereas the same happens with the corresponding evaporation enthalpy. Thus, we may conclude that the polymer–water interaction degree as well as the evaporated water fraction is quite similar for the various samples. Therefore, we would expect a similar level of Tg plasticization. Despite that, the quaternization effects dominate here on the polymer mobility.

A last comment on DSC refers to Figure 6b. Therein, upon dehydration, the PVP exhibits a clear glass transition step; meanwhile, on the other hand, within all quaternized samples the glass transition step has vanished. The effect suggests that the dehydration of PVPQ and PVP_Q1-3 resulted to extremely rigid structures. Upon removal of water molecules from the systems, the free volume decreases severely, whereas additionally, in the quaternized systems, dense crosslinking of the polymer chains is formed. Both parameters should be responsible for the ‘elimination’ of free polymer mobility. Another qualitative observation coming in to support to the latter, refers to the slopes (baselines) in Figure 6b. Comparing to PVP in the quaternized samples, the overall slope reduces. This is equivalent to the reduction of the heat capacity, c_p_; the dependence from temperature. The result can be rationalized considering the reduced fraction of mobile segments that additively contribute to the transport of heat. Similar recordings have been demonstrated in polymer nanocomposites, wherein the attractive polymer–filler interactions become dominant and eliminate the free polymer mobility [55,56]. From a methodological point of view, we should report that *c*_p_ is better represented by measurements in or close to equilibrium, namely, by step-scan or temperature modulation DSC. Interestingly, these effects seem independent from the modification degree; however, they are in accordance to the similar amount of evaporated water.

**Figure 7:**
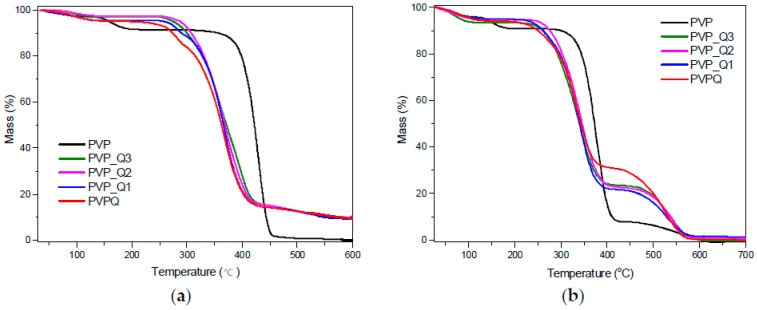
TGA measurements of PVP, as made, and the quaternized derivatives (**a**) under an N_2_ atmosphere and (**b**) under a flow of air.

The thermal stability of PVP and its quaternized derivatives was studied under both inert and oxidative conditions, Figure 7. Under inert conditions, two mass loss steps are observed. A small initial mass loss is recorded at low temperatures (up to 150 °C), which is attributed to the loss of adsorbed water. This is in agreement with the water evaporation observed by DSC (scan 3, Figure 6a). Then, the main degradation event that relates to the thermal degradation of the polymers is recorded between 300 and 400 °C for the quaternized polymers (maximum degradation rate around 365 °C) and 400–450 °C for PVP (maximum degradation rate around 420 °C). All quaternized polymers exhibit similar behavior with the two polymers containing the highest amount of quaternized units (PVPQ and PVP_Q1), demonstrating slightly lower stability compared to the two others. When it comes to air atmosphere, PVP behaves similarly by degrading at a higher temperature (between 350 and 400 °C). For the quaternized derivatives, an additional degradation step is observed between 500 and 600 °C. It can be assumed that the degradation products formed under O_2_ (oxides), which differ from the ones formed under N_2_, are more stable between 400 and 500 °C and result in a second mass loss between 500 and 600 °C.

Overall, the thermal stability of PVP decreases with quaternization. It has been documented that modification by quaternization causes a decrease in the thermal stability of the polymer, as it becomes susceptible to Hofmann elimination due to the ammonium groups. Indeed, the Hofmann elimination occurs when quaternary ammonium salts are exposed to high temperatures; the reaction yields an alkene, a tertiary amine and a low molecular weight compound specific for the counterion (HI) [59]. Nevertheless, the quaternized PVP derivatives exhibit significant thermal stability.

### 3.3. UV-Vis Spectroscopy

**Figure 8:**
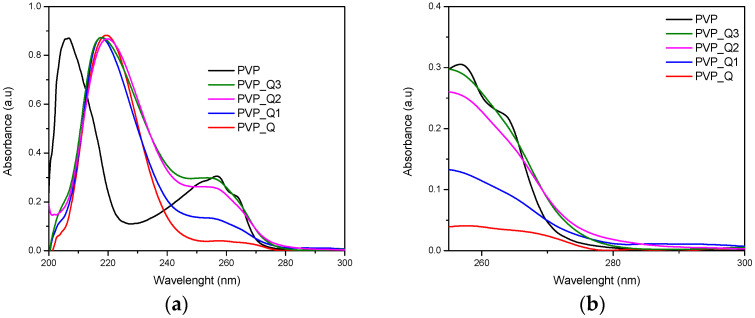
The UV-Vis spectra of PVP and the quaternized derivatives in methanol. (**a**) Full spectra. (**b**) Zoom in the 255–300 nm region.

The UV-Vis spectra of PVP and the partially quaternized derivatives are shown in Figure 8. Two well-defined UV bands, a strong one at 206 nm and a weaker one at 257 nm, are observed for the non-quaternized polymer. These bands are associated with n→π* transitions of N atoms with unshared electron pairs. However, in the case of the quaternized derivatives, an important shift from 206 to 217 nm was observed; this shift is associated with a bathochromic effect that occurs when the absorption wavelength shifts to longer wavelengths, indicating the formation of the pyridinium cation [23]. Moreover, it is observed that with increasing quaternization degree the intensity of the weaker band in the 260 to 280 nm region is decreased, probably due to the bonding of the nitrogen’s lone pair electrons. The decreased absorbance of the PVPQ derivatives in this region is in accordance with the band gap reduction, as shown below.

**Figure 9:**
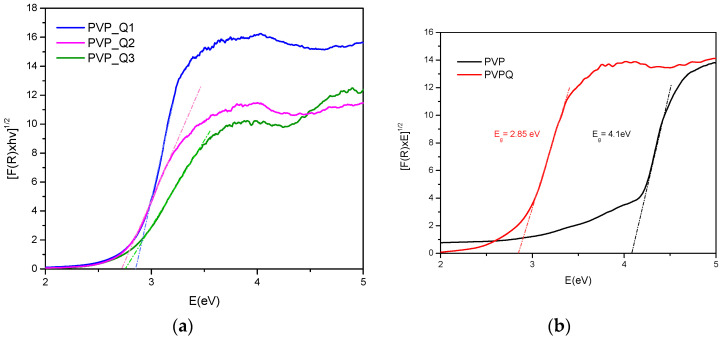
Kubelka–Munk plots of (**a**) PVP (Q1-Q3) and (**b**) PVP and PVPQ.

Diffuse reflectance spectra (DRS) in the solid-state were also recorded between 200–800 nm. DRS calculates the absorption, which corresponds to the electron transition from the valance band to the conduction band in order to determine the bandgap of materials. The Kubelka–Munk plots of PVP and PVPQ derivatives are presented in Figure 9. The inter band electronic transitions and the absorption spectrum of all polymers represents a strong and broad absorption feature in the UV region. The bandgap values are determined from the intersection of the extrapolation of the linear part of the plots to the x-axis, indicating energy. A considerable red-shift of absorption edge in higher wavelength numbers for all quaternized polymers is observed. This shift is attributed to the reduction of the energy gap, which it is achieved for all quaternized polymers, and can be observed in Figure 9, where the absorption functions (F × E)^1/2 =^ f(E) with F = (1 − R)^2^/2R corresponding to Kubelka–Munk function are presented. In Table 2, the significant difference in the energy gap between the quaternized and non-quaternized polymers is proved.

As clearly observed, the optical bandgap is reduced from 4.1 eV for the non quaternized PVP to 2.85 eV for the fully quaternized PVPQ. The reported band gap values for PVP and PVPQ (See Table 2) correspond to the energy gaps between the higher occupied molecular orbital (HOMO) and the lower unoccupied molecular orbital (LUMO) of the corresponding polymers. More specifically, the band gap of a molecular material, i.e., the difference between its valence and conduction band, corresponds in molecular orbital theory terms to the gap between HOMO and LUMO energy levels of the molecule (the macromolecule in the case of PVP and PVPQ). 

The optical bandgap (Eg) was found to decrease similarly in the partially quaternized polymers; however, it is without a well-defined relation toward the degree of quaternization in the monitored conditions. Nevertheless, a decrease can be explained for all polymers by the fact that charge transfer complexes (CTCs) in the host polymer were formed due to the quaternization of nitrogen. As a result, the lower energy transitions will be enhanced, leading to the observable optical bandgap changes [60].

Quaternization of the aromatic nitrogen by nucleophilic alkylation offers an alternative way to introduce positive charge into the backbone and provides opportunities to enhance the electron delocalization and the molecular properties of these polymers [33]. Consequently, the quaternized polymers exhibit a smaller energy gap; it should be mentioned that partial quaternization may contribute to the E_g_ reduction even more due to the random positioning of the positive charges and thus electron distribution.

### 3.4. Solvatochromic Study

The solvation effects occurring in the solutions of polymers are of high importance, as their study can reveal important information regarding key solvent–polymer interactions controlling the properties and macroscopic behavior of polymer solutions [61,62]. Studying these interactions experimentally has various limitations, many of which can be overcome using suitable probe molecules, which are sensitive to minute changes in their solvation microenvironment (cybotactic region) [63,64,65,66,67]. Herein, we employ the intensely sensitive solvatochromic betaine of Reichardt [68] in order to unravel the role of hydrogen bonding in solutions of PVP and PVPQ in binary solvent mixtures consisting of H_2_O and DMSO. Two main criteria of choice of these two solvents were considered; firstly, the solubility of the polymers and secondly, the involvement of protic and non-protic solvents in the investigated mixtures. Water can act both as a hydrogen bond donating (HBD) as well as a hydrogen bond accepting (HBA) solvent, whereas DMSO is an HBA solvent exhibiting considerable HBA and Lewis basicity. In general terms, water molecules are expected to efficiently create H-bonds with the N atoms of PVP and PVPQ involving lone pairs of electrons, whereas DMSO is anticipated to gather around/solvate the quaternized pyridinium entities of PVPQ. In addition to these basic specific and non-specific solvent–polymer interactions, important solvent–cosolvent interactions are of high importance, as DMSO and H_2_O efficiently form complexes when mixed; this happens at different extents depending on the molar ratio between the two solvents [69]. Table 3 shows the fourteen solvent ratios used as well as the recorded λmax in the presence of PVP and PVPQ. The λmax was measured three consecutive times and the average was noted.

As Reichardt’s betaine can act as an indicator of solvent polarity in DMSO/H_2_O mixtures (the preferential solvation of Reichardt’s betaine in DMSO/H_2_O mixtures has been examined thoroughly in the past [68]) alterations of the solvation effect in solutions of any of the polymers PVP or PVPQ in DMSO/H_2_O mixtures are expected to be detectable through the solvatochromism of Reichardt’s betaine. 

**Figure 10:**
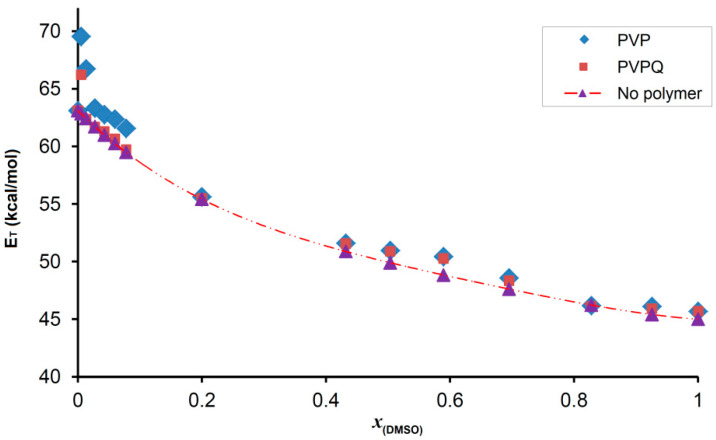
Plots of the measured Reichardt’s betaine charge-transfer energies in DMSO/water mixtures involving PVP and PVPQ, and absence of polymer versus the molar fraction of DMSO.

**Figure 11:**
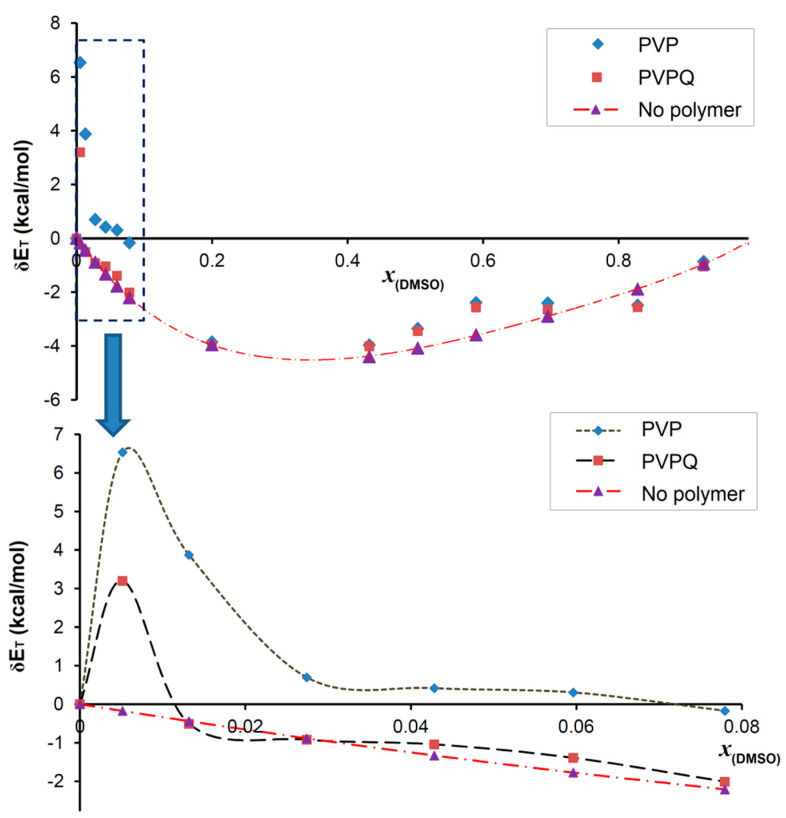
Plots of δE_T_ were observed in DMSO/H_2_O mixtures involving PVP and PVPQ, and the absence of polymer versus the molar fraction of DMSO. Top: full DMSO fraction range; bottom magnification of the bracketed DMSO fraction range 0–0.08.

Even though the solvatochromic shift patterns for solutions of PVP and PVPQ in DMSO/H_2_O mixtures appear to be very similar to those observed in the absence of polymer (see plots of Figure 10 and Figure 11 and Table 4) important deviations appeared in the water-rich regions of the plots. Specifically, in the case of PVP, δE_T_ (PVP) differences as large as 6 kcal/mol were observed. These large differences are obviously connected to the presence of the polymer (PVP). As water is very prone to H-bond to N atoms of PVP in low DMSO molar ratios (x_DMSO_ < 0.08), these large deviations from linearity are attributed to synergistic solvation effects encompassing the formation of complexes of the type PVP^…^HOH^…^RB (where RB corresponds to Reichardt’s betaine). The obtained microenvironment is sensed by Reichardt’s betaine, as highly polar, hence the large solvatochromic shifts. 

Similar effects are observed in the case of PVPQ, nevertheless, they appear to be attenuated when compared to PVP. This confirms the role of water-pyridine H-bonding in the observed solvation effect, as PVPQ contains a few non-quaternized N-atoms (i.e., N atoms with lone pairs of electrons prone to H-bonding with water molecules). Along with the remaining DMSO fraction range 1 > x > 0.08, the differences observed are less important, implying that the role of DMSO is less significantly altered in the presence of each of the polymers, PVP and PVPQ.

## 4. Conclusions

In this work, a series of new quaternized PVP polymers were synthesized and fully characterized. Characterization with FTIR and NMR essentially contributed to the understanding of the structure and composition (quaternization degree) of the fully (PVPQ) and partially (PVP_Q1-3) quaternized derivatives. Regarding the thermal behavior, as-made PVP as well as the quaternized PVPs exhibit single glass transition steps, with Tg systematically increasing and the heat capacity change decreasing with the modification. This suggests the transformation to a gradually more rigid matrix. In all cases the matrices are plasticized by similar amounts of hydration water (ambient), while the quaternization dominates on the hindering of the polymer chains diffusion. The thermal stability decreases with quaternization, due to the susceptibility of the quaternized polymers to Hoffman elimination reactions. Nevertheless, PVPQ and PVP_Q1-3 exhibit satisfactory thermal stability (up to 300 °C). When it comes to UV-vis absorbance, the quaternization reaction resulted in a red shift of the λmax from 206 to 217 nm for all PVPQs. Moreover, as an outcome of the quaternization, the optical energy gap of PVP was significantly reduced from 4.1 eV to 2.74–2.85 eV for PVPQ derivatives, which could be indicative of the potential of these materials in semiconducting and optoelectronic applications. Finally, the solvation behavior of the polymers was assessed in binary solvent mixtures of DMSO and water, employing a solvatochromic model dye, namely Reichardt’s betaine. The impact of solvent polarity versus solvent basicity was proven to play an important role in the solvation of the studied quaternized and non PVPs. More in-depth studies on the solvatochromism of these novel polymers are in progress, broadening the understanding of the solvation behavior and chemical bonding that goes along with the optical properties related to the polymers’ chemical structure. 

## Figures and Tables

**Table 1:** Characteristics of the polymers discussed in this study.

Sample	Calculated Degree of Quaternization (%)	Tg (°C)
PVP	0	86
PVP_Q3	35	86
PVP_Q2	46	98
PVP_Q1	85	101
PVPQ	100	114

**Table 2:** Bandgap values (Eg) of the quaternized and non-quaternized polymers.

Sample	Eg (eV)_Indirect
PVP	4.1
PVP_Q3	2.83
PVP_Q2	2.75
PVP_Q1	2.74
PVPQ	2.85

**Table 3:** Solvent systems and the respective RB’s absorption maxima in the visible region in the presence of PVP and PVPQ.

DMSO: H_2_O Ratio	PVP (nm)	PVPQ (nm)
100:1	635	634.5
98:2	629	631.5
95:5	628	629
90:10	597	600
85:15	575	577
80:20	569	570
75:25	562	562.5
50:50	515	514
25:75	471	485.5
20:80	465	478
15:85	462	473
10:90	458	470
5:95	434.5	465
2:98	417	438

**Table 4:** Results of the solvatochromic study of solutions of PVP, PVPQ, and absence of polymer in binary mixtures of DMSO and H_2_O involving Reichardt’s betaine as a solvatochromic indicator.

xDMSO	E_T_ (PVP) kcal/mol	E_T_ (PVPQ) kcal/mol	E_T_ (NP *) kcal/mol	δE_T_ (PVP) ^‡^ kcal/mol	δE_T_ (PVPQ) ^‡^ kcal/mol	δE_T_ (NP) ^‡^ kcal/mol
1.000	45.7	45.7	45.0	0.0	0.0	0.0
0.926	46.1	45.9	45.4	−0.9	−1.0	−0.9
0.828	46.2	46.1	46.2	−2.5	−2.6	−1.9
0.695	48.6	48.3	47.6	−2.4	−2.6	−2.9
0.590	50.4	50.3	48.8	−2.4	−2.6	−3.6
0.504	51.0	50.9	49.9	−3.4	−3.4	−4.1
0.432	51.6	51.6	50.9	−4.0	−4.0	−4.4
0.200	55.6	55.5	55.4	−3.9	−4.0	−4.0
0.078	61.6	59.7	59.5	−0.2	−2.0	−2.2
0.060	62.4	60.7	60.2	0.3	−1.4	−1.8
0.043	62.8	61.3	61.0	0.4	−1.0	−1.3
0.027	63.3	61.7	61.7	0.7	−0.9	−0.9
0.013	66.7	62.4	62.4	3.9	−0.5	−0.4
0.005	69.5	66.2	62.8	6.5	3.2	−0.2
0.000	63.1	63.1	63.1	0.0	0.0	0.0

* NP: Νo polymer; ^‡^ δE_T_ (PVP), δE_T_ (PVPQ) and δE_T_ (NP); the difference between measured charge transfer energy of Reichardt’ betaine measured in PVP and PVPQ, and in absence of polymer, respectively, and the ideal (linear dependence) Reichardt’ betaine charge-transfer energy are all measured in DMSO/water mixtures.

## Data Availability

The data presented in this study are available on request from the corresponding author.
